# Coherent multidimensional photoelectron spectroscopy of ultrafast quasiparticle dressing by light

**DOI:** 10.1038/s41467-020-16064-4

**Published:** 2020-05-06

**Authors:** Marcel Reutzel, Andi Li, Zehua Wang, Hrvoje Petek

**Affiliations:** 1 Department of Physics and Astronomy and Pittsburgh Quantum Institute, University of Pittsburgh, Pittsburgh, Pennsylvania 15260 USA; 2 Present Address: I. Physikalisches Institut, Georg-August-Universität Göttingen, Göttingen, Germany

**Keywords:** Electronic properties and materials, Surfaces, interfaces and thin films

## Abstract

Depending on the applied strength, electromagnetic fields in electronic materials can induce dipole transitions between eigenstates or distort the Coulomb potentials that define them. Between the two regimes, they can also modify the electronic properties in more subtle ways when electron motion becomes governed by time and space-periodic potentials. The optical field introduces new virtual bands through Floquet engineering that under resonant conditions interacts strongly with the preexisting bands. Under such conditions the virtual bands can become real, and real ones become virtual as the optical fields and electronic band dispersions entangle the electronic response. We reveal optical dressing of electronic bands in a metal by exciting four-photon photoemission from the Cu(111) surface involving a three-photon resonant transition from the Shockley surface band to the first image potential band. Attosecond resolved interferometric scanning between identical pump–probe pulses and its Fourier analysis reveal how the optical field modifies the electronic properties of a solid through combined action of dipole excitation and field dressing.

## Abstract (web summary)

Strong pulses of light can drive materials into nonequilibrium states with distinct physical properties. Here the authors observe the changes in copper’s electronic properties as intense optical fields dress the band structure and quasiparticle mass.

## Introduction

Space-periodic arrangement of lattice ions in crystalline solids defines the ***k***-momentum dispersion of electronic quasiparticle bands. Light can interrogate such bands by stimulating electric dipole transitions. When a time-periodic optical field interaction exceeds all other perturbations, particularly at an optical resonance, however, it can also modify the system eigenstates. Then a quantum system is modified (dressed) by an optical field, it becomes a time crystal with novel, field-dependent electronic properties^1^. High-optical field control, which ultimately enables the rich physics of high-harmonic and attosecond pulse generation^2^, then motivates the exploration of the light-wave electronics in solids^3–9^. Optical fields with designed electric field strength ${\cal{E}}$, and frequency *ω*_*l*_ can manipulate and control the quasiparticles in solids. Recent goals and achievements include light-induced superconductivity^10^, high-order nonlinearities^3,6^, the creation of Floquet topological bands in quantum materials^9,11–19^, and photoinduced phase transitions^20,21^.

**Fig. 1:**
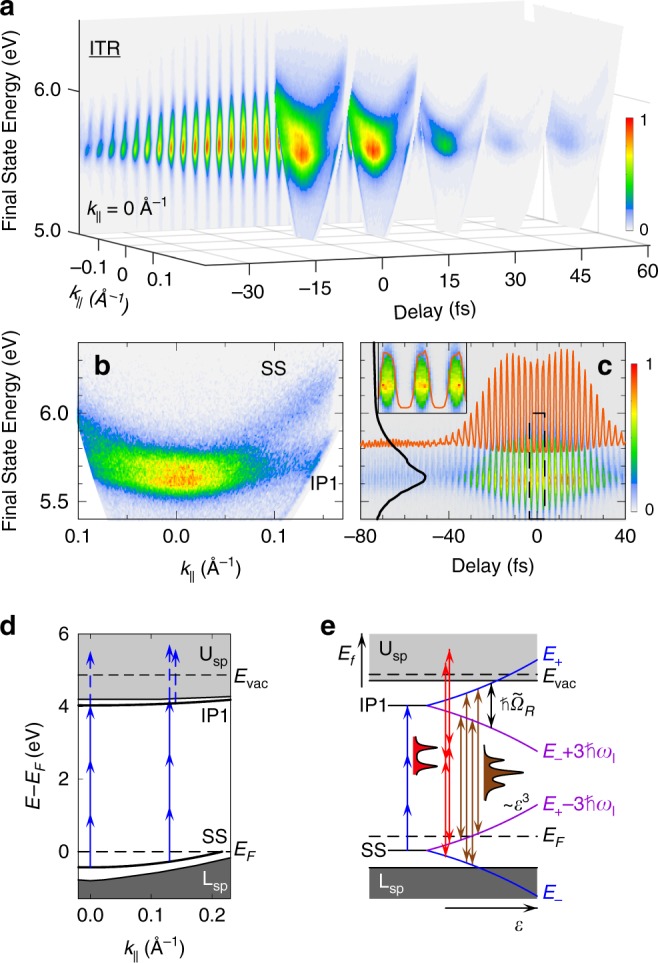
Coherent photoelectron spectroscopy of optical dressing. **a** Three-dimensional ITR-4PP movie of the three-photon resonant IP1←SS transition (*ħω*_*l*_ = 1.54−eV). Each frame of Supplementary Movie 1, from which the data is taken, records the photoelectron signal counts vs. *E*_*f*_ (relative to the Fermi level, *E*_F_), and *k*_||_ in ~100 as steps (sample movie frames are shown in 15 fs intervals). The back panel shows a cross section through the movie for *k*_||_ = 0 Å^−1^. **b** Time-integrated 4PP spectrum showing the SS and IP1 bands for resonant excitation at *k*_||_ = 0 Å^−1^. **c** Selection of data from **a** for *k*_||_ = 0 Å^−1^ showing an *E*_*f*_(*τ*)-interferogram; the profiles show I2PC trace for *E*_*f*_ = 5.65 eV (orange) and a 4PP spectrum for *τ* = 0 fs (black); the inset expands the *E*_*f*_(*τ*)-data around *τ* = 0 fs (the dashed box). The dressing is evident in the dip in I2PC intensity at *τ* = 0 fs that is caused by the subfemtosecond splitting of the interference fringes, when the pump and probe fields are in-phase and $\cal{E}$(*τ*) is maximum, but not in the *τ* = 0 fs *E*_*f*_-profile. **d** The unperturbed surface-projected band structure of Cu(111). The SS and IP1 bands (black lines) in the surface-projected band gap between the lower and upper *sp*-bands, L_*sp*_ and U_*sp*_ (gray shading), are coupled by the three-photon resonant laser field (blue); the fourth photon induces the photoemission (dashed blue). The excitation is resonant at *k*_||_ = 0 Å^−1^ producing one peak with enhanced 4PP yield in **b**; the different *m*_eff_ of the SS and IP1 bands, however, cause their detuning for increasing *k*_||_ and consequent detection of two bands. **e** The optical dressing of the SS and IP1 bands: the increasing electric field $\cal{E}$ (right arrow) causes AC Stark effect shifts of the SS and IP1 bands to *E*_±_ (blue), while the Floquet effect replicates *E*_±_ at integer multiples of *nħω*_*l*_ *are* (violet). The optical dressing is revealed by Mollow-Triplet structures in the resonant coherent polarizations at 3*ω*_*l*_ (brown arrows), and Autler–Townes doublets in the nonresonant coherent polarizations at 2*ω*_*l*_ (red arrows).

Under perturbative conditions, light fields ${\cal{E}}\left( \tau \right)$ can excite multiquantum transitions from dispersive Bloch bands that are defined by the crystal structure, with their eigenstates specified by their momentum ***k***. An intense ${\cal{E}}\left( \tau \right)$ field, however, can perturb the system by causing Rabi flopping to occur between the optically coupled bands at frequencies $\tilde \Omega _R$ that can become comparable to that of the excitation field, *ω*_*l*_ (ref. ^13^): the electronic bands are said to be dressed when the optical field amplitude and periodicity shifts their energies, and replicates them by integer photon quanta through the AC Stark and Floquet processes (Fig. 1e)^9,11^. The optical dressing is thus marked by Autler–Townes (AT) splitting of solid-state bands into a new eigenstates *E*_±_ (ref. ^22^) separated by the generalized Rabi frequency

1 $$\hbar \tilde \Omega _R\left( {\tau ,{\boldsymbol{k}}} \right) = \hbar \sqrt {{\mathrm{\Omega }}_R^2(\tau ) + \Delta ^2({\boldsymbol{k}})},$$

where $\hbar \Omega _R(\tau ) = \mu _{{\mathrm{eff}}}{\cal{E}}^n(\tau )$ is the Rabi frequency of a *n*-photon transition, and $\hbar \Delta \left( {\boldsymbol{k}} \right)$ is the ***k***-dependent detuning of Bloch bands ($\hbar$: the Planck constant). Moreover, we assume the dipole moment, $\mu _{{\mathrm{eff}}}$, to be independent of ***k*** based on approximately symmetric photoemisson intensity distributions (with respect to + and – parallel momentum, *k*_***||***_), and *k*_***||***_-independent linear photoemission spectra of the initial band^23^.

Here, we apply intense ultrafast optical fields to a metal surface, a solid-state plasma, whose high electron density defines its quantum optical characteristics: attosecond screening and femtosecond electronic dephasing^24–26^. The ground and excited surface electronic bands of several metals like copper exist in projected band gaps that decouple them from stronger bulk interactions^25^ (Fig. 1d), making them inviting targets for probing their quantum optics by ultrafast multiphoton photoemission (mPP) spectroscopy and many-body theory^25–30^. We dress the surface electronic structure of Cu(111) with ~20 fs IR laser pulses by exciting the three-photon resonant transition from the occupied Shockley surface (SS) to the first image potential (IP1) band, and probe the coherent field interaction by further exciting photoelectrons into the photoemission continuum by interferometric-time-resolved multiphoton photoemission (ITR-mPP) spectroscopy (Fig. 1). The dressing and probing field ${\cal{E}}$(*τ*) combines identical, phase correlated pump–probe pulse pairs to excite four-photon photoemission (4PP). Advancing their delay (phase) in Δ*τ* ~ 100 as steps defines the optical fields with subfemtosecond precision, and simultaneously imaging photoelectron energy, *E*_*f*_, and parallel momentum, *k*_||_, distributions records *E*_*f*_(*k*_||_,*τ*)-movies of coherent polarizations excited in the sample; the movies record the field-induced quasiparticle dressing at the interface between the perturbative and non-perturbative light–matter interactions (Fig. 1a, Supplementary Movie 1). We explore how the optical field modifies the crystal-defined band structure on a sub-optical cycle timescale.

## Results

### Ultrafast quasiparticle dressing probed by coherent mPP

The ${\cal{E}}$(*τ*) field with *ħω*_*l*_ = 1.54−eV excites the three-photon resonant transition from the SS to the IP1 band of Cu(111) at *k*_||_ = 0 Å^−1^. Different band dispersions of SS and IP1, which are defined by their band masses $(m_{SS,}m_{IP1})$, however, detune the resonant energy by $\hbar \Delta ( {k_{||}}) = \hbar ^2/2({m_{{\mathrm{SS}}}^{ - 1} - m_{{\mathrm{IP}}1}^{ - 1}})k_{||}^2$. The time-integrated 4PP spectrum in Fig. 1c confirms the SS and IP1 resonance^29,31^; such optical phase-integrated measurement, however, obscures their dressing, which, is encoded in the phase-resolved *E*_*f*_(*k*_||_,*τ*)-movies that reveal the electric field strength-dependent response (Fig. 1a, Supplementary Movie 1). To reveal the dressing, we first extract the ${\cal{E}}\left( \tau \right)$-dependent 4PP intensity for *k*_||_ = 0 Å^−1^ ($\hbar \Delta = 0$ eV) by plotting the 2D *E*_*f*_(*τ)* data and, its time profile (1D interferometric two-pulse correlation (I2PC)) showing the photoelectron counts vs. *τ* at *E*_*f*_ of the IP1←SS resonance (Fig. 1c). If the optical field ${\cal{E}}\left( \tau \right)$ with approximately a Gaussian time profile generated the 4PP signal by only exciting dipole transitions, the I2PC trace in Fig. 1c would follow ${\cal{E}}$^8^(*τ*) dependence, and therefore, sharply peak at *τ* = 0 fs (refs. ^30,32^), but instead, it has a local minimum and retarded maxima at *τ* ≈ ± 15 fs, which portend the optical field dressing.

### Fourier analysis of femtosecond field dressing of resonantly excited surface bands

**Fig. 2:**
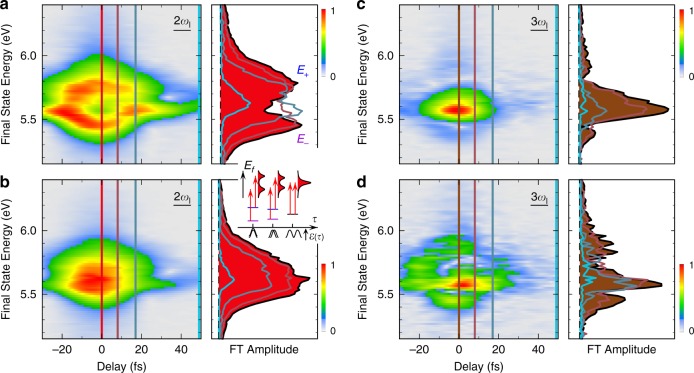
Femtosecond ${\cal{E}}$(*τ*) field undressing of the surface bands. IFT spectra of the 2*ω*_*l*_- **a**, **b** and 3*ω*_*l*_-polarization **c**, **d** responses at *k*_||_ = 0 Å^−1^ for excitation with high (top) and low (bottom) optical fluence. At high fluence and *τ* = 0 fs, in **a** and **c**, the AT doublet and the MT triplet structures are visible, respectively. As the instantaneous ${\cal{E}}$(*τ*) decreases with increasing *τ*; however, the dressed bands converge to undressed ones within ~15 fs (cf. inset). At low fluence, ${\cal{E}}$(*τ*) is sufficient to drive the 4PP process, but its dressing is minimal. Energy line profiles taken for selected delay points (color coded) are shown for each measurement in the right. The inset shows how the dressing depends on the instantaneous excitation fields, as *τ* is increased.

The *E*_*f*_(*k*_||_,*τ*)-movie, therefore, incorporates both the ${\cal{E}}\left( \tau \right)$-dependent band dressing and quantum excitation pathways, which are revealed by its Fourier analysis. Fourier transformation (FT) of the 2D time-domain data in Fig. 1c resolves the induced polarization fields in the sample that oscillate at 1–4*ω*_*l*_ frequencies to produce the 4PP signal^30^; in Fig. 2, we present the 2D-Fourier-filtered time-domain spectra of coherent polarizations oscillating at 2*ω*_*l*_- and 3*ω*_*l*_-frequencies, which reveal the dominant pathways for how these coherences contribute to the 4PP signal, and thereby reveal the dressing. As detailed in Supplementary Note 3, the Fourier filtering involves the FT of the 3D ITR-4PP data to resolve the 1–4*ω*_*l*_ frequency components that contribute to the signal, followed by the selective inverse Fourier transformation (IFT) of each.

The 2*ω*_*l*_ and 3*ω*_*l*_ IFT spectra show peak doubling and tripling, respectively, near *τ* = 0 fs when the optical field amplitude is the largest (Fig. 2). The 3*ω*_*l*_-polarization represented by brown arrows in Fig. 1e reports how the optical field dresses the resonant bands causing components at 3*ω*_*l*_ and $3{\upomega}_l \pm {\tilde{\mathrm{\Omega }}}_R$ frequencies to appear; such coherent polarizations produce Mollow-Triplet (MT) structures in gas-phase optical spectra^22^. By contrast, the nonresonant 2*ω*_*l*_-polarization, represented by red arrows in Fig. 1e, shows how the three-photon IP1⟵SS resonance dresses the interacting bands by revealing their AT doubling, when they are projected into the *E*_*f*_-continuum. The 2*ω*_*l*_- and 3*ω*_*l*_-spectra in Fig. 2, thus reveal the coherent subfemtosecond responses of the electronic bands to the dressing field. The 2*ω*_*l*_-spectrum shows that when *τ* = 0 fs and therefore, when ${\cal{E}}\left( \tau \right) = {\cal{E}}_{\mathrm{pump}}\left( t \right) + {\cal{E}}_{\mathrm{probe}}(t + \tau )$ is maximum, at resonance the AT doublet splitting is $\hbar {\tilde{\mathrm{\Omega }}}_R\left( \tau \right) \approx 0.3\,{\mathrm{eV}}$, but as |*τ*| increases to ~15 fs, the instantaneous ${\cal{E}}$(*τ*) field drops to recover the single undressed resonance. The dressing occurs within each optical cycle of 2.7 fs as evidenced by the subfemtosecond splitting of the I2PC fringes near *τ* = 0 fs (inset of Fig. 1c), when constructive interference maximizes ${\cal{E}}$(*τ*). As expected for the AC Stark effect (Eq. (1)), the AT doublet structure disappears when ${\cal{E}}$(*τ*) amplitude is reduced by defocusing (Fig. 2b) or increasing *τ*. Thus, the dressing and undressing follows the instantaneous ${\cal{E}}$(*τ*) strength, and is faster than the Cu(111) surface IP1 and SS band dephasing, which occurs by carrier scattering^25^ on ~20 fs timescale^28,29^.

### Entanglement of in-plane momentum dispersion and photon dressing

**Fig. 3:**
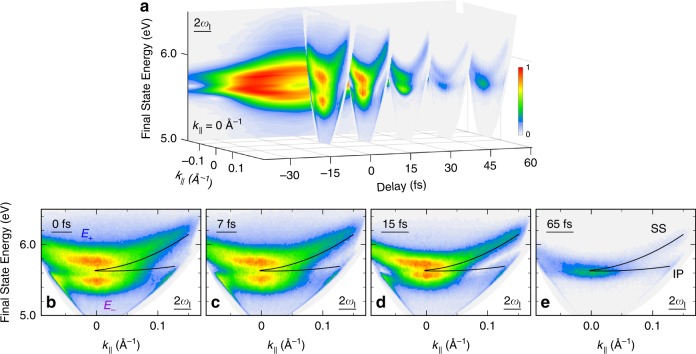
Real-time movie of the *k*_||_-dispersion-optical field entanglement. **a** IFT of the 2*ω*_*l*_-polarization component in Supplementary Movie 1 (also, the Fig. 1a data) provides a 3D presentation of the *E*_*f*_(*k*_||_,*τ*)-resolved AT doublet (the individual movie frames show the *E*_*f*_(*k*_||_)-spectra in 15 fs intervals; the entire filtered data are presented in Supplementary Movie 2). The back panel is the *τ*-dependence of the IFT *E*_*f*_(*k*_||_ = 0Å^−1^) profile, such as in Fig. 2a. **b**–**e** *E*_*f*_(*k*_||_)-resolved snapshots for *τ* = 0, 7, 15, and 65 fs showing undressing as *τ* is increased. As a reference, the black lines indicate dispersions of the undressed SS and IP1 bands obtained at *τ* = 100 fs from the unfiltered ITR-4PP data, where the dressing is undetectable.

A graphic feature that emerges from the ITR-4PP data on Cu(111) is that *k*_||_-dispersion and optical dressing are entangled by the generalized Rabi frequency (Eq.(1)), which depends both on ${\cal{E}}$(*τ*) and $\hbar \Delta ({k_{||}})$. The entanglement is directly evident in the 2*ω*_*l*_-IFT-spectral component, which we extract from the data in Supplementary Movie 1, and examine as a function of *k*_||_ and τ in Supplementary Movie 2. Supplementary Movie 2 conveys how the *E*_*f*_(*k*_||_)-dependent polarization spectra, change as *τ* is advanced in Δ*τ* ~ 100 as steps to define the total ${\cal{E}}$(*τ*) field. The selected movie images in Fig. 3, highlight how changing ${\cal{E}}$(*τ*) and *k*_||_-dispersion affect the AT doublet dressing of the coupled bands. For *τ* = 65 fs (Fig. 3e), the pump and probe fields do not overlap, so the instantaneous ${\cal{E}}$(*τ*) is minimum, and the spectra reproduce the undressed *k*_||_-dispersions of the IP1 and SS bands (black lines). By contrast, for $15\,{\mathrm{fs}} \lesssim |\tau |$ (Fig. 3b–d), the pump and probe fields add coherently, sufficiently increasing ${\cal{E}}$(*τ*) to cause the *k*_||_- and *τ*-dependent dressing, and thereby modify the band dispersions. As $\hbar \Delta ({k_{||}})$ increases, however, the bands detune from resonance and therefore dressing diminishes causing them to converge to the undressed ones. This entanglement of band dispersion and dressing causes the quasiparticle band masses to become *τ*-dependent. Thus, dressing can transform an electron into a hole band, potentially reversing the carrier transport in response to a femtosecond timescale optical field (cf. Supplementary Fig. 4), as could be evaluated by, for example, THz-optical photoemission spectroscopy^8^. Our coherent multidimensional movies show that light is a vector that can modify the electron mass, and physical properties that derive from it.

## Discussion

Besides the dressing and *k*_||_-dispersion entanglement and dephasing, however, we must consider the dressing from a solid-state perspective. A quasiparticle in a solid is defined by its many-body interactions, specifically how a solid-state plasma responds to its Coulomb fields on a subfemtosecond screening timescale^24,31^. Specifically, the IP1 band forms through the many-body screening response of a metal^26,32^, and therefore, the screening and dressing temporal responses must be interdependent, as further theoretical and experimental scrutiny should elaborate.

Finally, we compare our results to the femtosecond two-color angle-resolved photoemission spectroscopy of Floquet bands in a Bi_2_Se_3_ topological insulator (see Supplementary Note 5 for details) and the transition metal dichalcogenide, WS_2_, in polarization-dependent optical spectroscopy. Gedik and coworkers^16,33^ demonstrated how time-coincident nonresonant mid-infrared excitation introduces new Floquet bands, and causes Bloch–Siegert band shifts. By contrast, here, we report the optical dressing under three-photon resonant conditions where the Stark shift dominates. We report the optical phase*-*resolved, subfemtosecond, ${\cal{E}}$(*τ*)-dependent, entangled AT dressing through a nonlinear resonance between the equilibrium Bloch SS and IP1 bands. Such resonant dressing has only been reported for the fundamental semiconductor band gaps with all-optical ***k***-integrated techniques^12–14^.

Our 3D coherent photoelectron spectroscopy movies show that electronic bands are no longer defined just by the lattice potential, but applying a time-periodic external field introduces new Floquet bands, and causes shifts of nonlinearly excited resonant electronic bands through the AC Stark effect; specifically, breaking of the time-translation symmetry creates new nonequilibrium electronic structures within the solid state^1^, and opening of gaps between the time and space-derived bands entangles the space (*k*_||_) and time degrees of freedom, introducing frequency responses below that of the driving field. We stress that the interferometric-time- and angle-resolved mPP technique is particularly suited for recording of energy and momentum-resolved real-time movies of the field-dependent dressing of solids. Our multidimensional spectroscopy approach shows that optical dressing dynamics that may be obscured in time-integrated light intensity-dependent spectra, can become conspicuous by probing the induced coherences in electric field-dependent measurements. This experimental approach is applicable to all solid-state materials where momentum-dependent Floquet engineering might cause, for example, a time-dependent change of a material’s electronic band topology^9,17^. Moreover, the entanglement between dressing and band dispersions provides the means to optically control the physical properties that depend on the quasiparticle band mass on a few femtosecond timescale. Our study demonstrates that quantum optics of the oldest optical material, a metallic surface, along with those of other high electron density solids, no longer reflect the passive coherent responses to optical fields, but can be actively controlled with subfemtosecond precision by nonlinear interactions with judiciously phase and amplitude tailored optical fields.

## Supplementary information

Supplementary Information

Description of Additional Supplementary Files

Supplementary Movie 1

Supplementary Movie 2

## Data Availability

The data that support the plots within this paper and other findings of this study are available from the corresponding author upon reasonable request
