# Supplementary material for: Coherent multidimensional photoelectron spectroscopy of ultrafast quasiparticle dressing by light
Source: Nat Commun. 2020 May 6;11:2230. doi: 10.1038/s41467-020-16064-4 (PMC7203103; doi:10.1038/s41467-020-16064-4)
Supplement: Supplementary file 1 — Supplementary Information [file 41467_2020_16064_MOESM1_ESM.pdf]

Supplementary information to

Coherent multidimensional photoelectron spectroscopy of ultrafast quasiparticle dressing by light

Marcel Reutzel,[‡ &] Andi Li, Zehua Wang, and Hrvoje Petek[‡]

Department of Physics and Astronomy and Pittsburgh Quantum Institute, University of Pittsburgh, Pittsburgh, Pennsylvania 15260, USA

[&]present address: I. Physikalisches Institut, Georg-August-Universität Göttingen, Göttingen, Germany

email: marcel.reutzel@phys.uni-goettingen.de, petek@pitt.edu

The supplemental material contains a description of the $E_f(k_{||},\tau)$-resolved ITR-4PP movies, the experimental methods, the Fourier Analysis of the ITR-4PP data, fluence dependent ITR-4PP spectra, a two-band optical dressing model, and comparison of how dressing appears in ITR-4PP spectroscopy with to two-color angle resolved photoemission spectroscopy (ARPES) measurements.

Supplementary Note 1: $E_f(k_{||},\tau)$-resolved ITR-4PP Movies

Supplementary Movie **1** presents three-dimensional (3D) data streams of, photoelectron spectra displaying the photoelectron counts (color scale) as a function of the photoelectron energy E_f relative to E_F and $k_{||}$-momentum, and Supplementary Movie **2** displays the IFT of the $2\omega_l$-component of the data in Supplementary Movie **1**. The 4PP spectra of Supplementary Movie **1** are taken by advancing the pump-probe delay in $\Delta\tau\approx100$ as intervals to record the coherent polarization oscillations that capture the signal changes caused by the dressing of the electronic bands; the $\mathcal{E}(\tau)$ dependent coherent dressing is encoded in 4PP signal oscillations at the driving frequency ω_l and its higher harmonics involving,. At the top of the movie frames, the $\mathcal{E}(\tau)$ field envelopes are indicated schematically by a pair Gauss-profiles representing the scanning of the pump and probe fields. On the right side, the experimental temporal evolution of 4PP spectra at the $k_{||}$ = 0 $Å^{-1}$ reports the optical dressing of the electronic bands at the IP1←SS three photon resonance including the constructive and destructive interferences between the coherent polarizations excited in the sample. The 3D interferometric $E_f(k_{||},\tau)$-resolved data streams in Supplementary Movie **1** are the main observable of the ITR-4PP experiment.

Near the temporal overlap of the pump and probe pulses ($\tau\approx0$ fs), the 4PP $E_f(k_{||},\tau)$ signal intensity oscillates predominantly at the frequency of the excitation laser field reflecting the constructive and

destructive interferences as the phase (delay τ) between the pump and probe pulses is scanned. This modulates the total excitation field, $\mathcal{E}(\tau)$, and therefore, the surface band dressing that is captured in the photoemission signal. The field is sufficiently strong to dress the near resonant SS and IP1 bands only when the pump and probe pulses interfere constructively near $\Delta\tau\approx 0$ fs, but not when the pulses interact separately. This is because the generalized Rabi frequency that defines the dressing, has a $[\mathcal{E}(\tau)]^3$ dependence in a three-photon resonant process. In this raw ITR-4PP movie, the photon dressing causes strong modulation of the surface electronic bands, but it is not readily evident because the signal contains contributions from all coherent polarization components. Moreover, the 4PP signal is integrated over the entire interaction time, including contributions when the $\mathcal{E}(\tau)$ amplitude has diminished to below the strength that causes detectable dressing. Thus, resolving the dressing requires Fourier analysis of IFT-4PP data and examining how its Fourier components evolve as a function of pump-probe time delay. For delays well beyond the pulse temporal overlap, $\tau>30$ fs, polarization beating of the decaying coherences of the IP1 and SS bands continues, similar to that as reported for Cs/Cu(111) in Ref.[1]. This is caused by quantum interference independent on photon dressing and will be discussed in a future report. Its significance to this experiment is that it shows that the carrier dephasing, as reported in Ref.[2], occurs on a longer time scale than dressing.

The dressing affects the induced coherences excited in the sample so filtering the signal to analyze the specific frequency components helps to assign the dominant contributing processes. To reveal the dressing, as an example, the Supplementary Movie **2** selectively shows the temporal evolution of the $2\omega_l$-coherence, because it probes most directly how dressing affects the occupied band structure; the $E_f(k_{\|},\tau)$-dependent dressing of the surface bands is evident in appearance of an AT doublet in the nonresonant $2\omega_l$ polarization oscillations. The $k_{\|}$-dependent photon dressing in response to the

instantaneous field is evident in how the IP1←SS three photon resonance causes the SS band to split into two dressed components in the $E_f(k_{||})$-space as the pump-probe pulse temporal overlap increases the instantaneous electric field strength to a maximum at τ=0 fs. A detailed description of how dressing appears in the movie is presented in the main text, based on individual frames taken at selected τ (*cf.* Fig. 3). Again, for τ>30 fs, the signal is mainly probing the polarization beating of the remaining coherences involving the IP1 and SS bands. The Movie 2 is extracted from Movie 1 by applying Fourier analysis as discussed in more detail below in Supplementary Note 3.

Supplementary Note 2: Experimental Methods

Energy-, angle- (i.e., $k_{||}$), and time-resolved multidimensional ITR-4PP spectroscopy of a clean Cu(111) surface is performed in an ultrahigh vacuum chamber with a base pressure of <10^{-10} mbar. The single-crystal surface is prepared by repeated Ar^+ ion sputtering (20 min, 1500 V, 3 μA) and subsequent annealing (10 min, 800 K); surface quality is judged by the work function of the metal surface, negligible signal at the secondary electron cutoff (work function edge) compared with the surface band photoemission signal, as well as the dominant and sharp spectroscopic features from the SS and the IP bands. All experiments are performed at room temperature (295 K).

Femtosecond laser pulses in the near-IR region are generated with a noncollinear optical parametric amplifier (NOPA). The NOPA is pumped by the second harmonic of a Clark MXR Impulse fiber laser oscillator-amplifier system operating at a 1 MHz pulse repetition rate; pulse durations are typically ~20 fs as characterized by interferometric autocorrelation mPP measurements on the polycrystalline Ta sample holder that are described in more detail in Ref. [3]. 4PP spectra are excited by focusing *p*-polarized near-IR pulses at an angle of incidence of 45° onto the Cu(111) sample with an average power of ~100 mW, to achieve electric field strengths on the order of $10^9\ {}^{\mathrm{V}}\!/_{\mathrm{m}}$ (estimated from a calculated beam diameter of 100 μm). The electric field strength $\mathcal{E}$ on the surface can be adjusted by changing the focusing condition, i.e. by moving the focusing lens with respect to the surface to modify the laser fluence while keeping the average power, the pulse duration, and other experimental factors constant. The effects of possible sample damage and/or space-charge effect distortions of electron distributions are excluded due to long-term reproducibility of the mPP spectra on the same sample spot, as well as fluence-independent photoelectron peak energy and negligible excess broadening (*cf.* Supplementary Fig. 3). Energy- and angle-resolved photoelectron distributions

are detected by a hemispherical electron energy analyzer (Specs Phoibos 100) with a 2D position sensitive photoelectron counting detector. The electron analyzer measures the photoelectron energy-$k_{\|}$ dispersion in the optical plane.

The interferometric measurements are performed by generating identical pump-probe pulse-pairs with a self-made Mach-Zehnder interferometer and scanning their mutual delay (phase) in ~100 as steps by translating the length of one of its arms with a piezoelectric actuator. The primary output output of the Mach-Zehnder interferometer is directed into the sample, while the other output is used for the time-axis calibration; this is performed by passing the light through a monochromator to record the interference fringes between the pump and probe pulses at the center laser frequency[4]. The excitation pulses are focused onto the Cu(111) surface to excite 4PP and record $E_f(k_{\|},\tau)$ 2D images, which are combined into movies by stepwise advancing the pump-probe delay[5]. The excitation light dispersion is precompensated to achieve the minimum pulse durations of ~20 fs at the sample by multiple reflections from a pair of negative dispersion mirrors.

Supplementary Note 3: Fourier Analysis of the ITR-4PP data

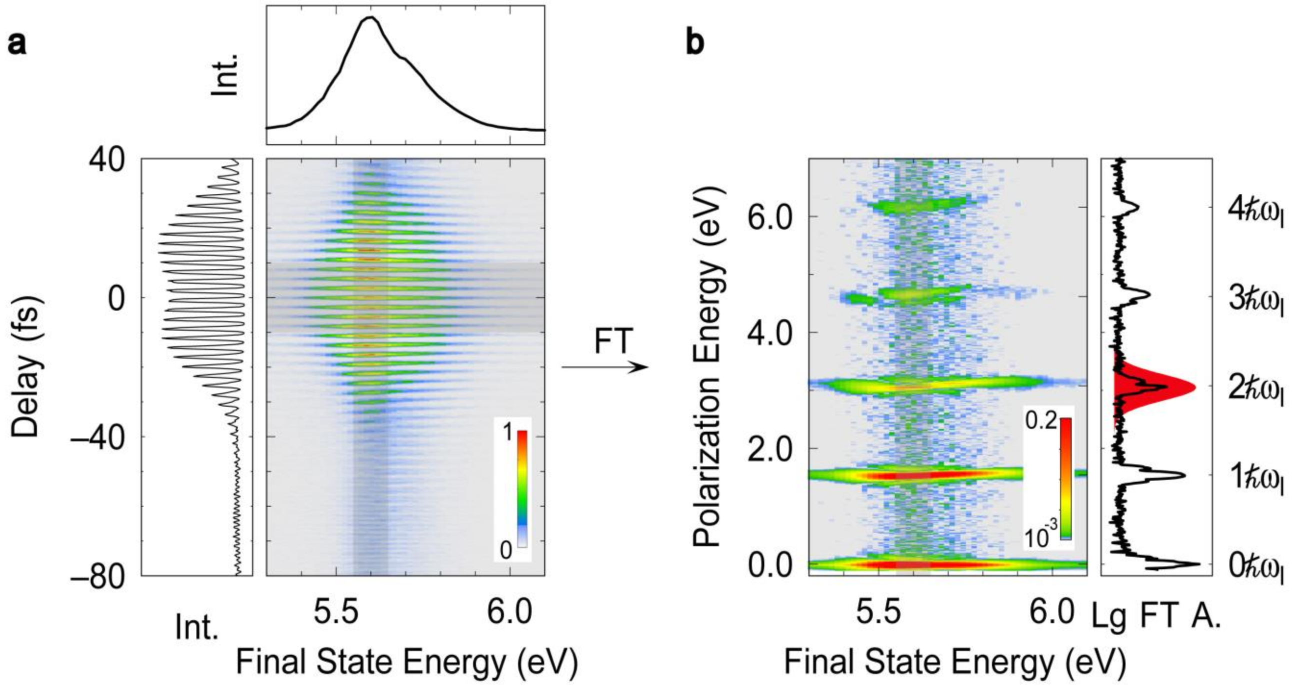

Supplemental Figure 1| Data processing of the multidimensional $E_f(k_{||},\tau)$-movies; the raw data for this figure are the same as shown in Fig. 1a of the main text, but presented here in a pedagogical format. **a** The normalized 4PP signal counts (color scale) are plotted as a function of $E_f = E - E_F$ and τ for $k_{||} = 0.0$ Å^{-1}. Interferometric two-pulse correlation plots (I2PC; left) and τ-dependent E_f-spectra (top) are extracted by integrating the 3D data sets over the E_f and τ ranges indicated by the grey shaded regions. **b** The 3D data is further analyzed by performing FT analysis with respect to τ to generate 2D-FT spectra that correlate polarization fields (coherences; the vertical FT axis) induced in the sample with E_f at which the excitation terminates (logarithmic color scale). The FT amplitudes have dominant components at the driving laser frequency $1\omega_l$ and its higher harmonics, indicative as well in the line profile taken along the polarization (FT) energy axis (gray region, logarithmic scale) at the 4PP energy of the IP1←SS three photon resonance. Inverse Fourier transforms (IFTs) of selected harmonics of the frequency space data are performed by multiplication of the FT data with Gauss-filter profiles [see the exemplary red profile at the $2\omega_l$-component] to isolate the selected frequency response for discussion of the temporal evolution of different orders of the induced polarization fields. The width of the filter function is chosen to be significantly broader than the peak width of the $n\omega_l$-component.

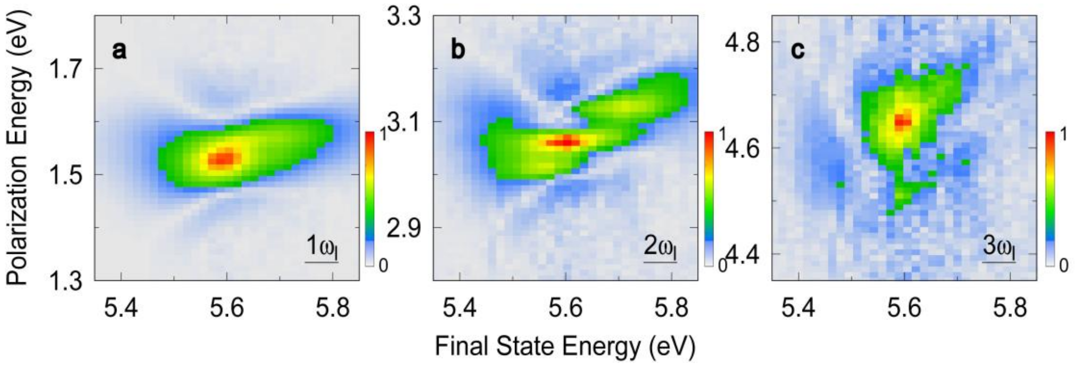

Supplemental Figure 2| Magnifications of the 2D-FT spectra shown in Fig. S1b. a-c 2D-FT spectra of the 1ω_l-, 2ω_l-, and 3ω_l-coherences showing the dressing induced structures (linear, normalized color scales).

ITR-4PP experiments collect 3D $E_f(k_{||},\tau)$-resolved movies of the coherent electron dynamics. From the 3D movies, we extract and plot the E_f- and τ-dependence of the photoelectron counts (color-scale) at a selected $k_{||}$ (Supplementary Fig. 1). Supplementary Figure 1a reproduces the experimental data (interferogram of 4PP for $k_{||}$ = 0.0 Å^{-1}) of the main manuscript (Fig. 1), with the primary focus on data processing and evaluation. Along the τ-axis, the 4PP yield is modulated by interferometric scanning between the identical pump and probe pulses, revealing delay (phase) dependent interferences of coherent polarizations excited in the sample; the modulation occurs at the optical cycle period (~2.7 fs) and its higher harmonics. The primary signature of the coherent response in the excitation process is the tilt of the $E_f(\tau)$-resolved interference fringes from the horizontal; a detailed analysis of structure of such interference fringes has been presented for a nonresonant 4PP process[3], where dressing does not occur. By generating line-profiles along the τ-axis (Supplementary Fig. 1a, vertical axis), I2PC traces are extracted that oscillate with the period of the driving laser frequency. Such I2PC traces report the coherences in optical excitation[4]; E_f- and thus eigenstate-resolved dephasing and decay parameters can be evaluated from these traces by simulation with an optical Bloch equation model[3, 4, 6]. In addition, τ-dependent E_f-4PP spectra reporting the occupied and unoccupied bands that contribute to the ITR-

4PP data can be generated by taking horizontal line-profiles (top of Supplementary Fig. 1a). Similar spectra obtained in 4PP spectroscopy without the time-frequency analysis integrate the signal over the interaction time obscuring the information on dressing.

By performing FT with respect to τ, we generate 2D-FT spectra, which correlate the polarization frequencies induced in the sample with the final state energy where the excitation terminates (Supplementary Fig. 1b); enlarged images of 2D-FT spectral components at harmonics of the driving frequency ω_l are shown in Supplementary Fig. 2. The FT analysis shows that the ITR-mPP signal has dominant density at the driving laser frequency $1\omega_l$ and its higher harmonics $n\omega_l$ that constitute a record of the coherent response of the sample in the mPP process. Depending on the polarization order, however, the 2D-FT amplitudes take on different profiles, being indicative of the different quantum coherences and dressing by the optical field that creates them (Supplementary Fig. 2a-c). In general, polarization fields at $n\omega_l$ are sensitive to quantum coherences between eigenstates of the system particularly when resonances occur at energies given by specific $n\hbar\omega_l$[3, 4]. Constructive and destructive interferences are evident in the multipeak structures recorded in the 2D-FT amplitudes; we attribute these multipeak structures to optical dressing of the surface bands. We note that 2D-FT spectra that portend dressing of the surface bands, show significantly more complex structures than what we reported for the nonresonant 4PP excitation of the surface bands in Ref. [3].

We explain the dressing of surface bands by examining the τ-dependence of the E_f-resolved polarization fields extracted by by performing inverse Fourier transform (IFT) of the filtered 2D-FT spectra in different orders of $n\hbar\omega_l$, as shown in Supplementary Fig. 1b. To obtain IFT spectra, such as in Fig. 2 and 3 of the main text, we first multiply the 2D-FT data with a window function, i.e. a Gauss-profile, that selects the desired polarization frequency region of one harmonic and excludes the other

harmonics (*cf.* red Gauss-profile in Supplementary Fig. 1b), and then perform IFT on thus filtered signal. The width of the Gauss filter function profile is chosen to capture the entire FT amplitude of the selected harmonic $n\hbar\omega_l$, without introducing unphysical signal distortions. This performs filtering of the ITR-4PP data in a selected frequency range of the coherent oscillatory response that participates in the nonlinear excitation process.

Supplementary Note 4: Fluence dependent Fourier filtered spectra

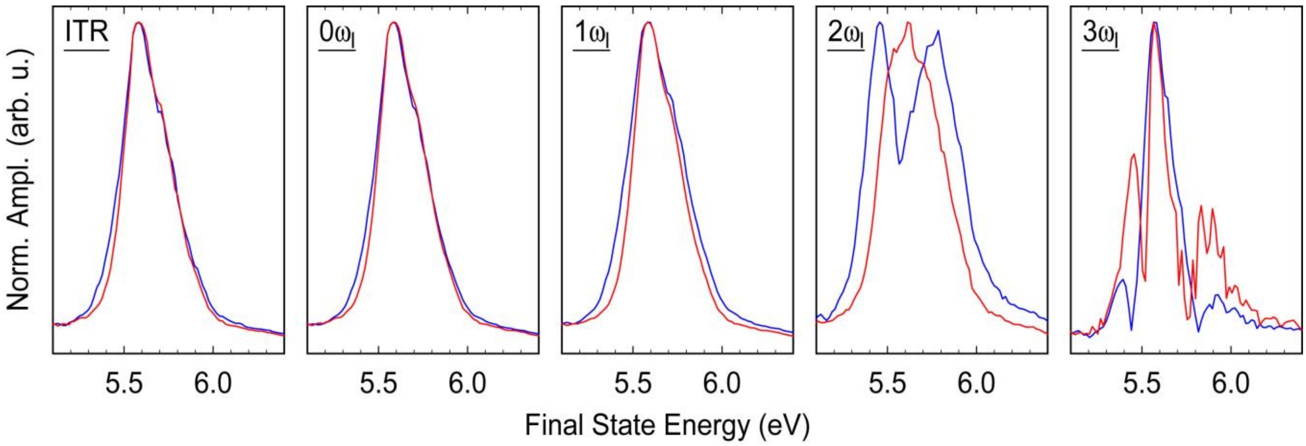

Supplemental Figure 3| The raw and Fourier filtered E_f-spectra for $k_{||}$=0.0 Å^{-1} and τ=0 fs. E_f-spectra showing the IP1←SS three photon resonance for τ=0 fs when the total excitation field is maximum. The spectra are taken from the same data as shown in Fig. 2 of the main text; the red and blue spectra correspond to small (*cf.* Fig. 2b/d) and large (*cf.* Fig. 2a/c) $\mathcal{E}$, respectively. Left to right: a 4PP spectrum taken from the raw ITR-4PP data; E_f-spectra generated via Fourier analysis of the ITR-4PP data filtered for contributions oscillating with $0\omega_l$ -$3\omega_l$-frequencies. While the $2\omega_l$- and $3\omega_l$-components show clear peak splitting into AT doublets and Mollow triplets at high $\mathcal{E}$, the raw data and the lower order components show negligible peak broadening and constant peak maxima, which imply negligible distortions of the spectra by experimental artefacts.

In the nonlinear ITR-4PP experiment performed in this work, the laser field is at the threshold where the field induced dressing starts to affect the measurement, but quantum dipole transitions still dominate the multi-photon process. More prosaically, the laser fluence is still sufficiently low so that undesired photoelectron space-charge distortion, and sample damage do not affect the measurements. The field strength dependent changes (splitting) of the $2\omega_l$- and $3\omega_l$-components and the lack thereof in the lower order components and the raw ITR-4PP data is only consistent with the dressing mechanism.

We identify the AC Stark and Floquet effects in the Fourier filtered ITR-4PP data by the observation of the increasing peak splitting of the AT doublet with increasing $\mathcal{E}$ (Supplementary Fig. 3 and Fig. 2 in the main text). By contrast, for the raw ITR-4PP spectra, we observe neither substantial peak broadening nor a shift in the maximum peak energy position, which could portend non-dressing artefacts affecting the data[7].

Supplementary Note 5: Two-Band Optical Dressing Model

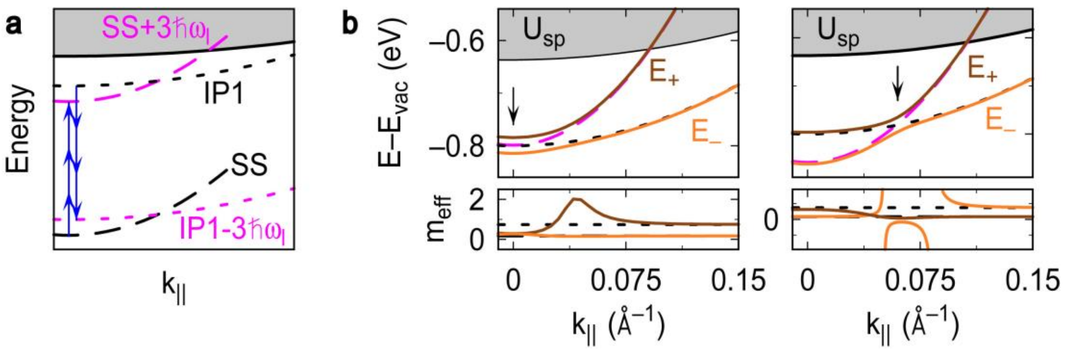

Supplemental Figure 4| Two-band model of photon dressing of parabolically dispersing bands. **a** Surface projected band structure of Cu(111), focusing on the $k_{||}$-dispersion of the SS and IP1 bands. Floquet bands projected up and down by three-photons are indicated in magenta, respectively. **b** $k_{||}$-dispersion and effective masses m_{eff} of the photon dressed bands (brown and orange) as calculated by a $\hbar\Delta(k_{||})$-dependent two-band model for two different resonance conditions. *Avoided crossing* occurs where the Floquet replica of the SS band, and the IP1 band (black) intersect at $k_{||}$ = 0.0 Å^{-1} (left) and 0.06 Å^{-1} (right) and split based on the AC Stark effect; the $k_{||}$-dispersion (top) and m_{eff} (bottom) of the dressed bands deviate from parabolic behavior of the pure states. The energy axis is given with respect to the vacuum level, E_{vac}, of Cu(111).

In the following, we briefly outline the calculated $k_{||}$-dependence of optical dressing for a two-band model system representing the resonant transition from SS to the IP1 band (Supplementary Fig. 4). More detailed descriptions of the underlying physics of dressing in atomic systems are available in standard textbooks on nonlinear and quantum optics, e.g., in Ref. [8]. The purpose of this modeling is to motivate how the entanglement of the optical dressing and $k_{||}$-dispersion transiently modulates the carrier masses, even converting electron into hole bands in a solid-state system.

In Supplementary Fig. 4a, we model the dressing by simulating the optical interaction for a two level system that participates in a three photon resonance. For linear light-matter interaction, the Bloch-Siegert effect can cause dressing in a nonresonant processes, but its modification of the electronic

bands their will be negligible compared to a nonlinear three-photon resonance. The nonresonant linear optical dressing of bands by combined Floquet, Stark, and Bloch-Siegert effects, described by Gedik and coworkers[9], is expected to be insignificant because the excitation light is strongly detuned from resonance, compared with the three-photon nonlinear resonant IP←SS transition. The unperturbed ground and the excited bands of the 2-band system stand for the parabolically dispersing SS and IP1 bands with their respective binding energies E_{SS} and E_{IP1}; the $k_{||}$-dispersion of the unperturbed eigenstates is given by $E_{\mathrm{SS,IP1}}(k_{||}) = \frac{\hbar^2}{2m_e m_{\mathrm{eff}}} k_{||}^2$. Both, the binding energy $E_{\mathrm{SS,IP1}}$ and the respective effective masses m_{eff} of the unperturbed surface states are extracted from the ITR-4PP experiment when the pump-probe pulses are far from temporal overlap (τ≈100 fs), and consequently the dressing field is weak. When including optical perturbation, the SS and IP1 bands (Bloch bands) are dressed, and replica Floquet bands are expected at integer photon energy intervals. In Supplementary Fig. 4a, the perturbed Floquet bands in 3rd photon order are indicated in magenta. The photon energy defines where the unperturbed Bloch and Floquet bands cross, i.e., the $k_{||}$ where $\hbar\Delta(k_{||}) = 0$ eV.

When the perturbation by the external electric field $\mathcal{E}(\tau)$ is included, the interaction Hamiltonian is expressed as

$$H = \begin{pmatrix} E_{\mathrm{SS}}(k_{||}) & \mu_{\mathrm{eff}}\mathcal{E}(\tau)^3 \\ \mu_{\mathrm{eff}}\mathcal{E}^*(\tau)^3 & E_{\mathrm{IP1}}(k_{||}) \end{pmatrix},$$

where the cubic dependence of the electric field strength $\mathcal{E}(\tau)$ describes the dominant field perturbation in a 3-photon transition from the SS to the IP1 state (μ_{eff}: effective dipole)[10]. The diagonal matrix elements are the unperturbed surface band energies, and the off-diagonal element is the Rabi frequency $\hbar\Omega_R = \mu_{\mathrm{eff}}\mathcal{E}^3(\tau)$ of their optical coupling. Diagonalization of the Hamiltonian gives the new set of dressed eigenstates $E_{\pm}(k_{||})$, which are plotted in the upper panels of Supplementary Fig. 4b. Under the multiphoton resonant condition, $3\hbar\omega$, an *avoided crossing* of the bands occurs and the

new quasi-eigenstates can be obtained by diagonalizing the interaction Hamiltonian; this defines the dressed bands, $E_{\pm}(k_{||})$, with the AC Stark effect modifying the dispersion in proportion to $\mathcal{E}^3(\tau)$. The dressing modifies the $k_{||}$-dependent effective masses, $m_{\text{eff}} = \hbar^2/m_e \left(\frac{d^2E_{\pm}(k_{||})}{dk_{||}^2}\right)^{-1}$, as shown in Supplementary Fig. 4b. Depending on the dressing field and the resonance condition, the sign of m_{eff} in dressed bands can be reversed, with the effect of changing charge transport from an electron to hole type on a few femtosecond time scale.

Supplementary Note 6: Photon dressed bands probed in one-color ITR-mPP and two-color ARPES

Femtosecond two-color ARPES

In pioneering experiments, Gedik and coworkers[11, 12] described the excitation and detection of Floquet bands in femtosecond two-color ARPES experiments. On Bi_2Se_3, a topological insulator, they could resolve the series of Floquet replicas of the Dirac cone (DC) by driving the material with an IR pump pulse and subsequently probing the photon dressed band structure with a UV probe pulse (see sketch in Supplementary Fig. 5a). As pointed out in Ref.[11, 12, 13, 14], however, in two-color ARPES, Volkov bands can overlap the Floquet bands complicating the signal analysis. The Volkov process is the photon dressing of photoelectron states before they emerge from the surface (Supplementary Fig. 5b)[15]. The scattering between the Floquet and Volkov bands can further impede the clear distinction between the dominant physical process leading to Floquet replicas in photoemission spectra. Gedik and coworkers performed a two-color ARPES experiment, where they distinguished between the Floquet and Volkov bands based on the polarization of the excitation light (*s* or *p*), and the $k_{\|}$-direction (k_x or k_y) of the avoided crossings. Note that in a two-color ARPES experiment, there is no coherence between the pumping and probing process; the UV pulse detects Floquet bands at different energies in a phase integrated manner. Therefore, the coherent spectroscopy of optical dressing with optical cycle resolution, such as shown in Figs. 2 and 3 of the main text, cannot be obtained.

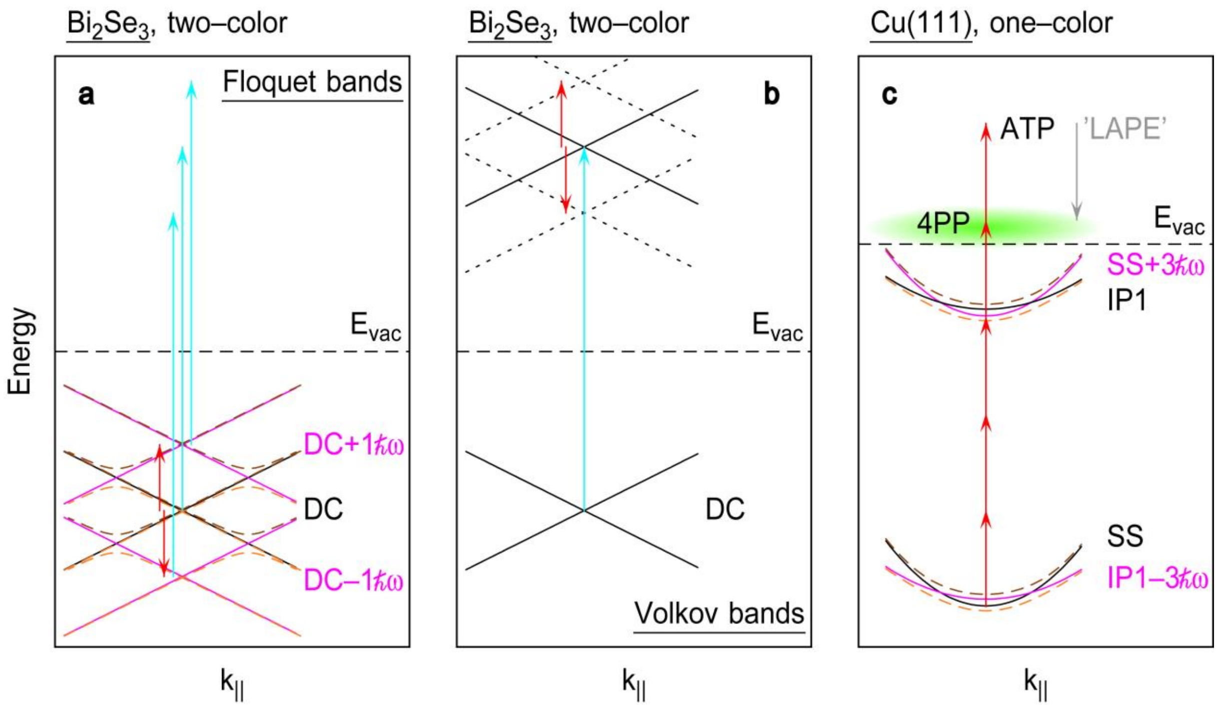

Supplemental Figure 5| Comparison of photon dressed bands probed by femtosecond two-color ARPES (Bi_2Se_3)[11] and one-color ITR-mPP [Cu(111)]. a/b Photon dressed bands involving linearly dispersing initial states, as probed by femtosecond two-color ARPES described in Ref.[11] (cyan: UV laser, red: IR laser, DC: Dirac cone). **a** Under irradiation with an IR pump laser, Floquet bands (magenta) form at energies separated by $n\hbar\omega_l$ from the Bloch band (black). Avoided crossings defined by the dressing field strength are observed for $k_{||}$ where bands of different photon order cross (brown and orange). An UV laser pulse can probe the dressed band structure by further excitation to above E_{vac}; the interactions by the IR and UV fields is incoherent. **b** In a parallel process, the UV probe pulse can excite photoemission, and before escaping from the surface, the photoemitted (free) electrons can be dressed by laser-assisted photoelectric effect (LAPE) to be detected as Volkov bands. In this case, no gap opening between the different order bands is expected. In a two-color IR-UV ARPES experiment, the Floquet and Volkov processes occur in the same order of photon interaction. **c** The possible occurrence of Floquet and Volkov processes is different in the case of the one-color experiment on Cu(111). The IR laser (red) builds up the Floquet series (magenta), in the process of exciting electrons from SS to above E_{vac} to induce photoemission in overall 4PP (green area). Higher order interactions can excite band replicas at larger energies to be observed as above threshold photoemission (ATP)[3, 16]. At $k_{||}$ where the Floquet replica SS+$3\hbar\omega_l$ (IP1-$3\hbar\omega_l$) and the Bloch band IP1 (SS) intersect, avoided crossings are expected based on the AC Stark effect (brown and orange). The photon dressed bands are subsequently probed above the vacuum level in overall 4PP (4th order process) and potentially ATP (5th order process). To have a significant contribution from 'LAPE', however, in a one-color process, one more photon interaction from ATP is necessary, as indicated by the grey arrow. Thus, in a one-color experiment, for LAPE to contaminate the 4PP signal would require the 4th and 6th order processes to terminate at the same final energy and have comparable excitation probabilities. Note that, for simpler visualization, the Floquet series is only shown in third-order of the photon field.

One-color ITR-mPP

In the following, we contrast the commonly performed two-color ARPES experiments with the one-color ITR-mPP method. A schematic excitation diagram of the one-color mPP scheme is shown in Supplementary Fig. 5c; the undressed SS and IP1 bands of the Cu(111) surface are shown in black. In the one-color experiment, the IR frequency builds up the Floquet series (magenta in Supplementary Fig. 5c), causes the AC Stark splitting of the resonant bands, and ultimately probes the dressing by promoting excitation through the dressed bands to excite 4PP. We note that in the one-color experiment, the measurements have to be performed with *p*-polarized light to detect photoelectrons from the surface bands of Cu(111).

The photon dressed bands reported in Fig. 2 and 3 of the main text arise due to the resonant coupling of the IP1←SS transition. The resulting dressing is evident in the gap opening described by the AC Stark effect; the dressed states $E_{\pm}$ are probed by further photoemission above E_{vac} in overall 4PP (green ellipse in Supplementary Fig. 5c). In addition, as we[3, 16] and others[15, 17] have reported, intense IR excitation of metal surfaces leads to above threshold photoemission (ATP), where photoelectrons absorb more photons then necessary to overcome E_{vac} (Supplementary Fig. 5c). Under our experimental conditions, this ATP yield decreases by at least one order-of-magnitude for each increasing non-linear order m ($m \geq 5$) under our experimental conditions [3, 16]. In one-color experiment, such ATP is a prerequisite for detection of LAPE through a Volkov process, which could potentially overlap with a Floquet based process. We can exclude the significant contribution of Volkov bands, however, as follows: Volkov bands, created by LAPE, would have to build up by photon dressing of free electrons (grey arrow in Supplementary Fig. 5c). For LAPE to contribute to 4PP signal electrons would need to undergo a transition from an ATP level above it in an overall 6th-order process (m=6),

which is expected to be more than two-orders of magnitude weaker than the direct 4th-order 4PP. We can thus exclude Volkov states (LAPE) from contributing to the ITR-4PP experiment.

The Fourier analysis of the one-color ITR-4PP data facilitates the extraction of the ultrafast dressing dynamics, which is otherwise hidden in the time-integrated spectroscopy data shown in Fig. 1b of the main text. Thus, to observe similar physics in a two-color experiment one would have to perform a three pulse pump-pump-probe experiment, where the two pumps are of the same color and are phase correlated, and the probe pulse reports the populations that are generated by the coherent two-pump interactions.

References

1. Ogawa S, Nagano H, Petek H. Phase and energy relaxation in an anti-bonding surface state: Cs/Cu(111). *Phys Rev Lett* **82**, 1931-1934 (1999).

2. Ogawa S, Nagano H, Petek H, Heberle AP. Optical dephasing in Cu(111) measured by interferometric two-photon time-resolved photoemission. *Phys Rev Lett* **78**, 1339-1342 (1997).

3. Reutzel M, Li A, Petek H. Coherent Two-Dimensional Multiphoton Photoelectron Spectroscopy of Metal Surfaces. *Physical Review X* **9**, 011044 (2019).

4. Petek H, Ogawa S. Femtosecond Time-Resolved Two-Photon Photoemission Studies of Electron Dynamics in Metals. *Prog Surf Sci* **56**, 239-310 (1997).

5. Cui X, Wang C, Argondizzo A, Garrett-Roe S, Gumhalter B, Petek H. Transient Excitons at Metal Surfaces. *Nat Phys* **10**, 505-509 (2014).

6. Weida MJ, Ogawa S, Nagano H, Petek H. Ultrafast interferometric pump-probe correlation measurements in systems with broadened bands or continua. *J Opt Soc Am B* **17**, 1443-1451 (2000).

7. Passlack S, Mathias S, Andreyev O, Mittnacht D, Aeschlimann M, Bauer M. Space Charge Effects in Photoemission with a low repetition, high intensity femtosecond laser source *J Appl Phys* **100**, 024912 (2006).

8. Boyd R. *Nonlinear Optics*. Academic Press (2008).

9. Sie EJ, Lui CH, Lee Y-H, Fu L, Kong J, Gedik N. Large, valley-exclusive Bloch-Siegert shift in monolayer WS2. *Science* **355**, 1066-1069 (2017).

10. Hosseini SA, Goswami D. Coherent control of multiphoton transitions with femtosecond pulse shaping. *Phys Rev A* **64**, 033410 (2001).

11. Mahmood F*, et al.* Selective scattering between Floquet-Bloch and Volkov states in a topological insulator. *Nat Phys* **12**, 306-310 (2016).

12. Wang YH, Steinberg H, Jarillo-Herrero P, Gedik N. Observation of Floquet-Bloch States on the Surface of a Topological Insulator. *Science* **342**, 453-457 (2013).

13. Park ST. Interference in Floquet-Volkov transitions. *Phys Rev A* **90**, 013420 (2014).

14. Baggesen JC, Madsen LB. Theory for time-resolved measurements of laser-induced electron emission from metal surfaces. *Phys Rev A* **78**, 032903-032907 (2008).

15. Saathoff G, Miaja-Avila L, Aeschlimann M, Murnane MM, Kapteyn HC. Laser-assisted photoemission from

surfaces. *Phys Rev A* **77**, 022903 (2008).

16. Reutzel M, Li A, Petek H. Above-threshold multiphoton photoemission from noble metal surfaces. *Phys Rev B* **101**, 075409 (2020).

17. Sirotti F*, et al.* Multiphoton *k*-resolved photoemission from gold surface states with 800-nm femtosecond laser pulses. *Phys Rev B* **90**, 035401 (2014).
