# Supplementary material for: Coherent multidimensional photoelectron spectroscopy of ultrafast quasiparticle dressing by light
Source: Nat Commun. 2020 May 6;11:2230. doi: 10.1038/s41467-020-16064-4 (PMC7203103; doi:10.1038/s41467-020-16064-4)
Supplement: Supplementary file 2 — Description of Additional Supplementary Files [file 41467_2020_16064_MOESM2_ESM.pdf]

Description of Additional Supplementary Files

File Name: Supplementary Movie 1
Description: The movie presents three-dimensional data streams of, photoelectron spectra displaying the photoelectron counts (color scale) as a function of the photoelectron energy E_f relative to E_F and $k_{\parallel}$-momentum. Details are described in the Supplementary Note 1.

File Name: Supplementary Movie 2
Description: The movie displays the IFT of the $2\omega_l$-component of the data shown in Supplementary Movie 1. Details are described in the Supplementary Note 1.
